# 5-Oxo-1-[(2,3,6,7-tetramethoxy-9-phenanthrenyl)methyl]-L-proline Inhibits Hepatitis C Virus Entry

**DOI:** 10.1038/s41598-019-43783-6

**Published:** 2019-05-13

**Authors:** Lap P. Nguyen, Chorong Park, Trang T. D. Luong, Eun-Mee Park, Dong-Hwa Choi, Kang Min Han, Han N. Mai, Huu C. Nguyen, Yun-Sook Lim, Soon B. Hwang

**Affiliations:** 1 Laboratory of RNA Viral Diseases, Korea Zoonosis Research Institute, Chonbuk National University, Iksan, South Korea; 2 National Research Laboratory of Hepatitis C Virus, Hallym University, Anyang, South Korea; 3 Korea National Institute of Health, Cheongju, South Korea; 4 Graduate School of East-West Medical Science, Kyung Hee University, Yongin, South Korea; 5 Department of Pathology, Dongguk University Ilsan Hospital, Goyang, South Korea

**Keywords:** Molecular medicine, Gastroenterology

## Abstract

Hepatitis C virus (HCV) is the major causative agent of chronic liver diseases, including liver cirrhosis and hepatocellular carcinoma. The recent development of highly effective direct-acting antivirals (DAAs) has revolutionized the treatment of HCV patients. However, these DAAs are exorbitantly expensive for the majority of HCV patients worldwide. Moreover, these drugs still show genotypic difference in cure rate and have some resistant-associated variants. Tylophorine, a natural compound derived from *Tylophora indica* plants, is known to have anti-inflammatory and anti-cancerous growth activities. In the present study, we showed that two tylophorine intermediates, 5-Oxo-1-[(2,3,6,7-tetramethoxy-9-phenanthrenyl) methyl]-L-proline (O859585) and 2,3,6,7-tetramethoxy-9-phenanthrenecarboxylic acid (T298875), displayed anti-HCV activity with an EC_50_ of 38.25 µM for T298875 and 29.11~35.3 µM for O859585 in various HCV genotypes. We demonstrated that O859585 efficiently blocked HCV attachment by neutralizing free viral particles without affecting other stages of the HCV life cycle and interferon stimulation. O859585 interrupted binding between HCV E2 and CD81. Of note, co-treatment of O859585 with either interferon alpha (IFNα) or sofosbuvir exerted either an additive or synergistic antiviral activity in HCV-infected cells with no measurable effect on cell viability. Most importantly, O859585 in combination with IFNα and sofosbuvir exhibited synergistic effects on anti-HCV activity in primary human hepatocytes. Collectively, these data suggest that O859585 may be a novel antiviral agent for HCV therapy.

## Introduction

Hepatitis C virus (HCV) is one of the major causative agents of chronic liver diseases, contributing to 350,000 deaths annually^1,2^. HCV is an envelope virus that is approximately 55 nm in diameter. HCV belongs to the genus *Hepacivirus* within the family *Flaviviridae*. HCV has a positive-sense, single-stranded RNA genome that is 9,600 nucleotide in length and encodes a single polyprotein of 3,010 amino acids^3^. This single polyprotein is subsequently processed into three structural proteins (core, E1 and E2) and seven nonstructural proteins (p7, NS2 to NS5B)^4^. There are 7 major genotypes and more than 50 subtypes. Globally, genotype 1 predominates, contributing to 60–75% of cases^5^. There is no prophylactic HCV vaccine yet. However, several candidates for HCV vaccine are in early stage of clinical trials. Moreover, there is no completely effective therapy for chronic HCV infection. Until few years ago, chronic HCV has low success rates of treatment depending on genotypes. Pegylated interferon (PEG-IFN) and ribavirin (RBV) was mainly used to treat all genotypes of HCV. However, this therapy is being superseded by the advent of direct acting antivirals (DAAs). Currently, highly improved and simplified interferon (IFN)-free and ribavirin (RBV)-free regimens are available for HCV patients. These include NS3/4A protease, NS5B polymerase, and NS5A inhibitors, including simeprevir, sofosbuvir, ledipasvir/sofosbuvir, and daclatasvir^2,5^. However, cure rates of new DAAs vary depending on HCV genotypes, and exorbitant cost of DAAs is the major obstacle for the majority of HCV patients worldwide^6,7^. In addition, drug escape mutants occur due to low genetic barriers to resistance.

Natural products have been considered as sources of new anti-HCV drugs. Tylophorine, derived from *Tylophora indica* plants, has been revealed as a potential drug for cancer therapy because of its dramatic anti-cancer activity against various cancer cell lines^8^. In addition, tylophorine analogs such as antofine, DCB-3500, and DCB-3503 also show cancer growth inhibitory activities, especially for hepatocellular carcinoma^9,10^. We have previously demonstrated that tylophorine exerts a CycA2 inhibitory function and abrogates HCV replication^11^.

In the present study, we further showed that both O859585 and T298875, two precursors of tylophorine, markedly suppressed HCV propagation. Of note, O859585 exerted a stronger antiviral activity than T298875. We demonstrated that O859585 inhibited HCV infection at binding/attachment step of the HCV life cycle. HCV entry is a very complex process that involves a series of host entry and thus natural molecules of viral entry inhibitors may be particularly important for the treatment of HCV patients to minimize re-infection after liver transplantation^12^. Most importantly, O859585 in combination with either IFNα or sofosbuvir exhibited either an additive or synergistic anti-HCV activity, suggesting that O859585 may be a promising candidate for combination therapy for HCV patients.

## Results

### O859585 and T298875, tylophorine precursors, inhibit HCV propagation

**Figure 1:**
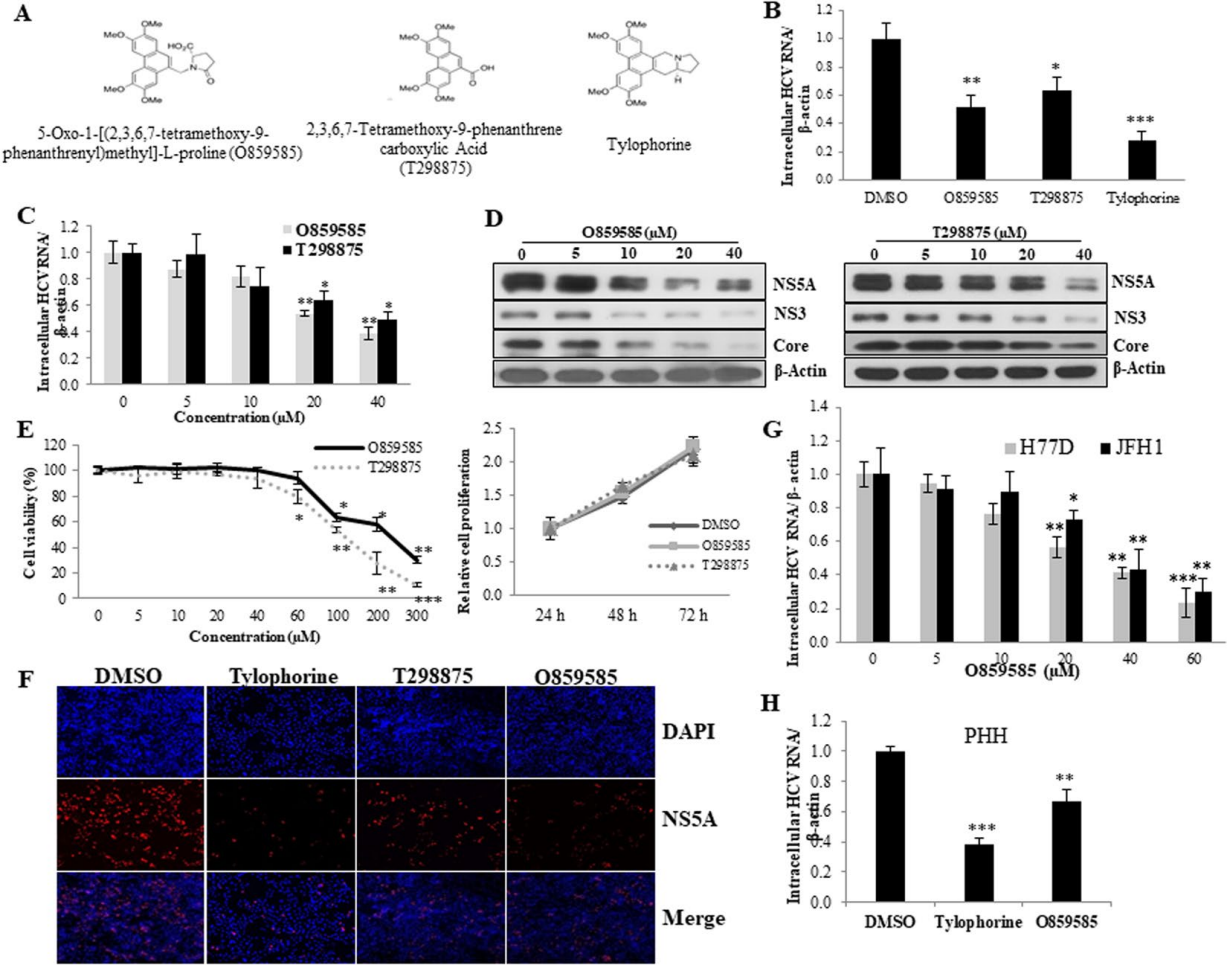
O859585 and T298875, tylophorine intermediates, inhibit HCV propagation. (**A**) Chemical structures of O859585, T298875, and tylophorine. (**B**) Huh7.5 cells were pretreated with either 20 µM O859585, 20 µM T298875, or 0.075 µM tylophorine for 1 h, and then infected with Jc1 for 4 h in the presence of the indicated chemicals. Cells were further cultured with fresh media containing each chemical. At 48 h postinfection, intracellular HCV RNA levels were quantified by qRT-PCR. (**C,D**) Huh7.5 cells were pretreated with various concentrations of either O859585 or T298875 for 1 h and then infected with Jc1 for 4 h in the presence of each chemical. Cells were further cultured with fresh media containing various concentrations of each chemical. At 48 h postinfection, intracellular HCV RNA (**C**) and protein (**D**) levels were quantified by qRT-PCR and immunoblot analysis, respectively. (**E**) (Left panel) Huh7.5 cells were treated with various doses of either O859585 or T298875 for 48 h and then cell toxicity was determined by using WST assay. (Right panel) Huh7.5 cells were treated with either DMSO (vehicle) or 29.65 µM O859585, or 38.25 µM T298875 for 1 h and then infected with Jc1 for 4 h in the absence or presence of each chemical. At the indicated time intervals after Jc1 infection, cell proliferation was determined by WST assay. (**F**) Huh7.5 cells were seeded on glass coverslips and treated with either DMSO (vehicle) or 0.075 µM tylophorine, 20 µM T298875, 20 µM O859585, respectively for 1 h and then infected with Jc1 for 4 h in the absence or presence of each chemical. At 48 h postinfection, cells were fixed in 4% paraformaldehyde and further incubated with NS5A antibody. Cells were counterstained with 4′,6-diamidino-2-phenylindole (DAPI) to label nuclei (blue signal). (**G**) Huh7.5 cells were pretreated with various concentrations of O859585 for 1 h and then infected with HCV derived from either genotype 1a (H77D) or genotype 2a (JFH1) for 4 h. At 48 h postinfection, intracellular HCV RNA levels were determined by qRT-PCR. (**H**) Primary human hepatocytes were pretreated with either DMSO, 0.075 µM tylophorine, or 20 µM O859585 for 1 h and then infected with Jc1 for 4 h in the presence of the indicated chemicals. Cells were further cultured with fresh media containing each chemical. At 48 h postinfection, intracellular HCV RNA levels were quantified by qRT-PCR. Data represent averages from three independent experiments for panel (B,C,G,H). The asterisks indicate significant differences (*p < 0.05; **p < 0.01; ***p < 0.001).

We have previously reported that tylophorine, the natural plant product, abrogates HCV replication^11^. In the present study, we explored the possible inhibitory functions of two precursors of tylophorine, O859585 and T298875 (Fig. 1A), in HCV replication. For this purpose, Huh7.5 cells were pretreated with either O859585 or T298875, or tylophorine and then infected with Jc1 in the presence of each chemical. At 48 h postinfection, intracellular HCV RNA levels were determined. As shown in Fig. 1B, both O859585 and T298875 significantly decreased intracellular HCV RNA levels. Tylophorine was used as a positive control. We further demonstrated that both O859585 and T298875 decreased intracellular HCV RNA levels in a dose-dependent manner (Fig. 1C). The half maximal effective concentration (EC_50_) of each chemical was 29.65 µM and 38.25 µM for O859585 and T298875, respectively. We next examined the effects of O859585 and T298875 on viral protein expression levels. Consistently, both O859585 and T298875 inhibited HCV protein levels in a dose-dependent manner (Fig. 1D). To investigate the side effect of chemicals on cell growth, the WST assay was employed. Figure 1E showed that both O859585 and T298875 exerted no cell toxicity up to 60 µM (Fig. 1E, left panel) and thus cell proliferation was not affected by these two chemicals (Fig. 1E, right panel). The half maximal cytotoxicity concentration (CC_50_) was ~214.44 µM and 157.97 µM for O859585 and T298875, respectively. We next investigated anti-HCV activities of tylophorine and its precursors by immunofluorescence assay. Consistent with previous report^11^, tylophorine strongly inhibited HCV propagation (Fig. 1F). We showed that both O859585 and T298875 also markedly suppressed HCV propagation. Of note, O859585 exerted stronger anti-HCV activity than T298875 (Fig. 1F). We therefore selected O859585 and investigated its effect on HCV replication using HCV genotype 1a (H77D) and 2a (JFH1). As shown in Fig. 1G, O859585 significantly decreased intracellular HCV RNA levels of both genotypes in a dose-dependent manner. Interestingly, anti-HCV activity of O859585 was stronger in genotype 1a than genotype 2a. The EC_50_ of O859585 for H77D and JFH1 was 29.11 µM and 35.35 µM, respectively. Finally, we used primary human hepatocytes to verify an anti-HCV activity of O859585. Consistently, O859585 suppressed intracellular HCV RNA levels in primary human hepatocytes (Fig. 1H). Tylophorine was used as a positive control^11^. Taken together, both O859585 and T298875 markedly suppressed HCV propagation. It was noteworthy that O859585 exerted stronger antiviral activity than T298875 in HCV-infected cells.

### O859585 specifically inhibits HCV infection at binding/attachment step

**Figure 2:**
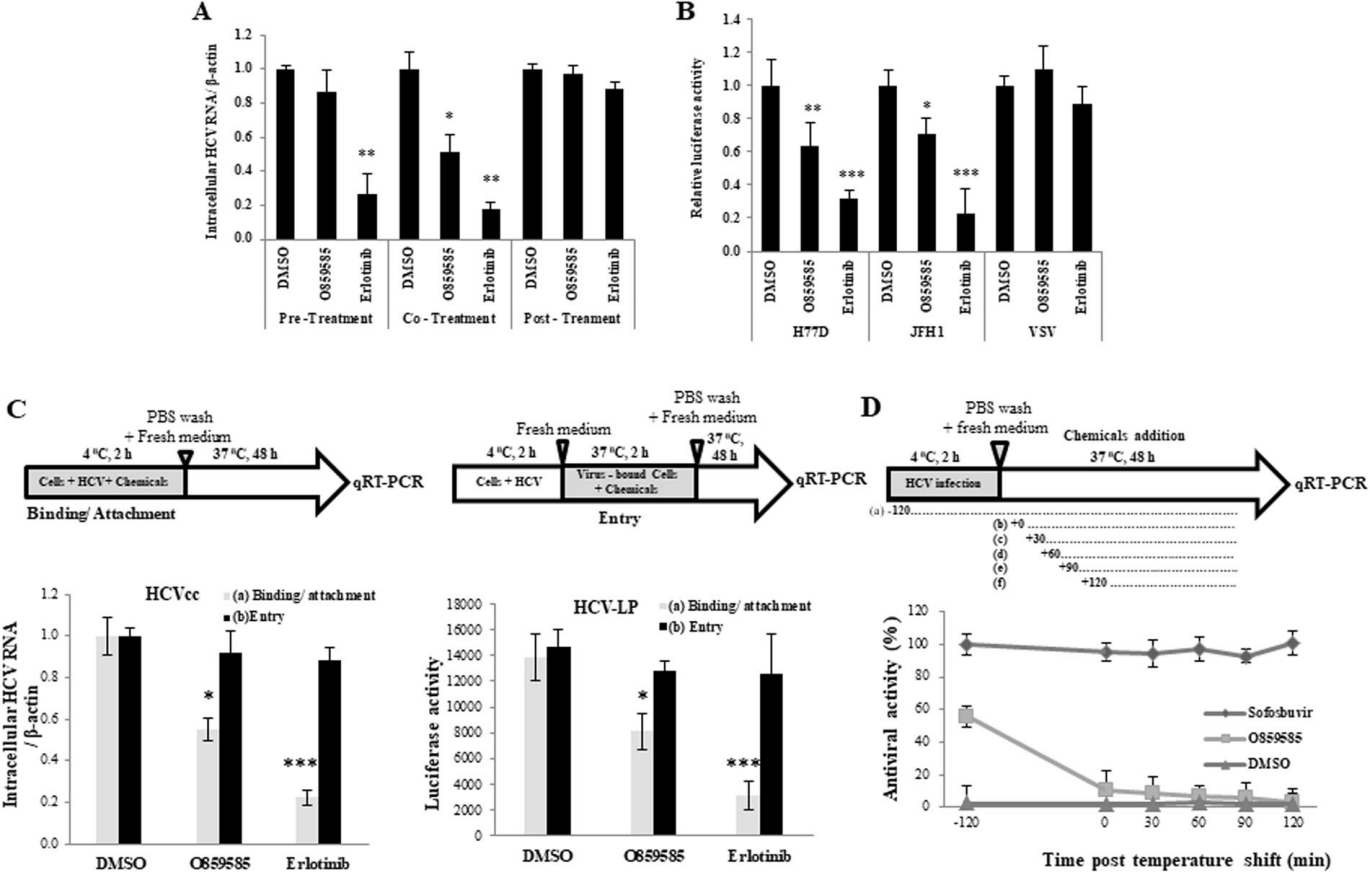
O859585 interupts HCV entry at binding/attachment step. (**A**) Huh7.5 cells were either pretreated with the indicated chemicals, washed twice with PBS, and then infected with HCV (pre-treatment), or infected with HCV in the presence of the indicated chemicals (co-treatment), or infected with HCV and then treated with the indicated chemicals (post-treatment) as described in Materials and methods. At 48 h postinfection, intracellular HCV RNA levels were determined by qRT-PCR. 0.1% DMSO (vehicle), 20 µM O859585, and 5 µM erlotinib were used for each experiment. (**B**) Huh7.5 cells were pretreated with DMSO, 20 µM O859585 or 5 µM erlotinib for 1 h. Cells were then infected with either VSVpp or HCVpp derived from either genotype 1a (H77D) or 2a (JFH1) for 6 h. At 48 h postinfection, viral entry was determined by luciferase activity. (**C**) For binding/attachment assay, Huh7.5 cells were incubated with either HCVcc (left panel) or HCV-LP (right panel) at 4 °C for 2 h in the presence of the indicated chemicals. The cells were washed with PBS and then bound HCV were determined either by analyzing RNA levels (left panel) or luciferase activity (right panel). For entry assay, Huh7.5 cells were incubated with either HCVcc (left panel) or HCV-LP (right panel) at 4 °C for 2 h in the presence of the indicated chemicals. The cells were washed with PBS and then temperature was shifted to 37 °C for 4 h. The cells were trypsinized and washed twice with PBS to remove free virions. Internalized HCV virions were indirectly determined either by analyzing RNA levels (left panel) or luciferase activity (right panel). (**D**) Huh7.5 cells were incubated with Jc1 for 2 h at 4 °C and then temperature was shifted to 37 °C. Each of 0.1% DMSO, 20 µM O859585, and 0.1 µM sofosbuvir was added at the indicated time points. At 48 h postinfection, antiviral activities in each time points were determined by analyzing HCV RNA levels. Data represent means ± SD of three independent assays. *p*-values are indicated by asterisks (*p < 0.05; **p < 0.01; ***p < 0.001).

To investigate which step of the HCV life cycle was targeted by O859585, we performed the time-of-addition assay as reported previously^1,13^. Huh7.5 cells were treated with chemicals and infected with Jc1 as indicated, and intracellular HCV RNA levels were determined. As demonstrated in Fig. 2A, neither pre-treatment nor post-treatment of O859585 suppressed HCV RNA levels. However, co-treatment of O859585 with viral infection revealed a significant reduction of HCV RNA level. To further investigate the possible involvement of O859585 in entry step of the HCV life cycle, we performed HCV pseudoparticle (HCVpp) entry assay. Huh7.5 cells were pretreated with the indicated chemicals and then infected with either HCVpp derived from either genotype 1a (H77D) or 2a (JFH1) or VSV pseudoparticles (VSVpp) in the presence of each chemical and then viral entry was analyzed. We showed that O859585 significantly suppressed HCVpp entry in both genotypes without affecting VSVpp entry (Fig. 2B). Erlotinib, an epidermal growth factor receptor (EGFR) inhibitor, is a well-known inhibitor of HCV entry and thus used as a positive control^14^. To further confirm these results, we performed time-of-addition entry assays using both cell culture grown HCV (HCVcc) and HCV like particle (HCV-LP) to distinguish between the binding/attachment and the entry/fusion steps. We first examined whether O859585 inhibited HCV infection at binding/attachment. Huh7.5 cells were infected with either HCVcc or HCV-LP in the presence of O859585 at 4 °C and then cultured for 48 h in the absence of inhibitor. To further investigate whether O859585 inhibited HCV infection at entry step of the HCV life cycle, Huh7.5 cells were infected with either HCVcc or HCV-LP in the absence of O859585 at 4 °C and then temperature was shifted to 37 °C, incubated for 2 h in the presence of inhibitor, and then cultured for 48 h. As shown in Fig. 2C, intracellular HCV RNA level was significantly decreased at the binding/attachment step but not at the viral entry step (left panel). Consistently, HCV-LP luciferase activity was significantly suppressed at the binding/attachment step (Fig. 2C, right panel). Erlotinib was used as a positive control. To further confirm these results, Huh7.5 cells were treated with O859585 at various time points. As shown in Fig. 2D, O859585 exerted the highest antiviral activity at the binding/attachment step and gradually decreased with time during entry step of the HCV infection. All these data indicate that O859585 specifically inhibits HCV infection at binding/attachment step of the HCV life cycle.

### O859585 blocks HCV entry by disrupting the binding between E2 and CD81

**Figure 3:**
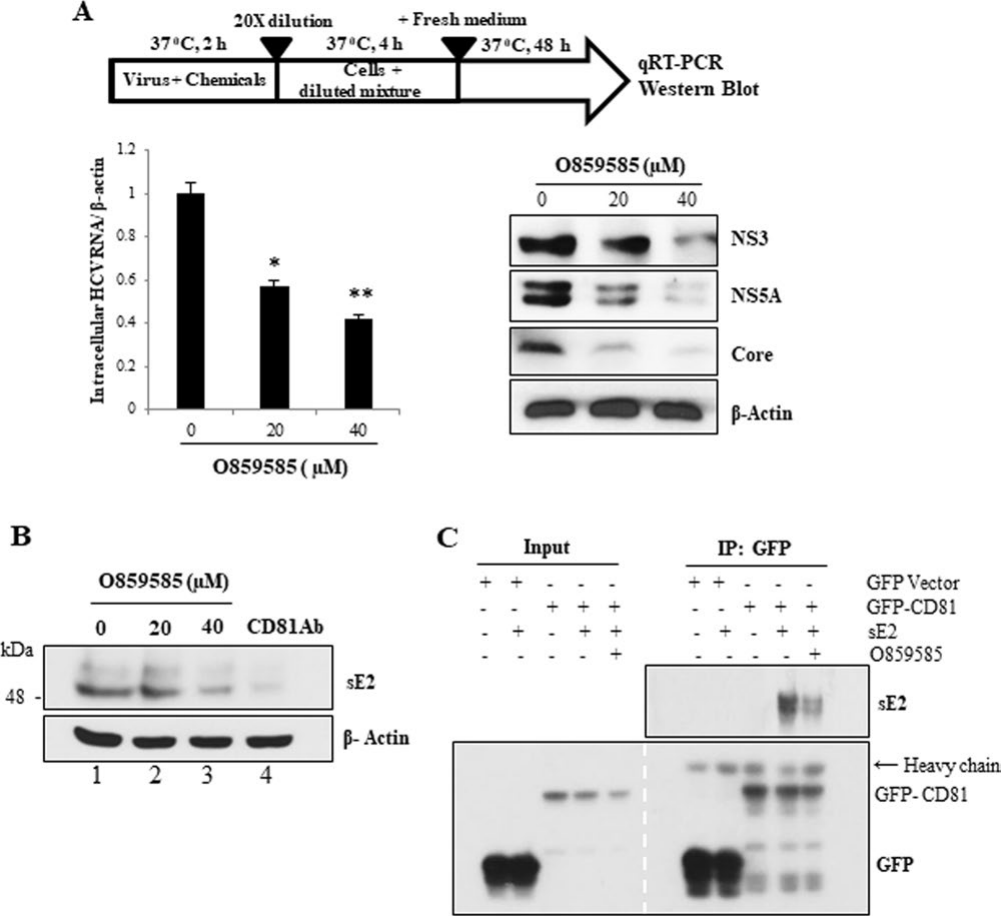
O859585 neutralizes HCV virions by disrupting E2 and CD81 binding. (**A**) HCV Jc1 virions were mingled with various concentrations of O859585 for 2 h at 37 °C. The mixture was diluted 20-fold to make ineffective concentration of O859585 and used to infect Huh7.5 cells. At 48 h postinfection, the viral RNA (left panel) and protein (right panel) levels were analyzed by qRT-PCR and immunoblot assay, respectively. Data are means of 3 independent experiments (*p < 0.05; **p < 0.01). (**B**) Huh7.5 cells were incubated for 4 h with sE2 protein derived from genotype 1a in the presence of various doses of O859585 or 1 µg/ml CD81 antibody. The cells were washed with PBS to remove unbound sE2 and then total cell lysates were immunoblotted with E2 antibody. (**C**) HEK293T cells were transfected with GFP-CD81. At 48 h after transfection, total cell lysates were incubated with sE2 in the absence or presence of 20 µM O859585. Total mixtures were immunoprecipitated with GFP and bound protein was immunoblotted with an anti-E2 antibody.

To further delineate the antiviral effect of O859585 on HCV, cell-free HCV virions were incubated with O859585 for 3 h at 37 °C and then mixture was diluted 20-fold to prevent any meaningful interaction of remained O859585 with the target cells^1,15^. Huh7.5 cells were incubated with diluted virus-chemical mixture. At 48 h postinfection, both viral RNA and protein levels were analyzed to evaluate the inhibitory activity of the chemical. As shown in Fig. 3A, O859585 significantly decreased HCV RNA levels (left panel). Consistently, HCV protein expression levels were markedly suppressed by O859585 (Fig. 3A, right panel). It is well-known that HCV E2 interacts with cellular CD81 to facilitate viral entry^16^. We therefore explored the possible involvement of O859585 in E2 and CD81 interaction. For this purpose, Huh7.5 cells were incubated with sE2 protein and various doses of O859585^17^. CD81 antibody was used as a positive control. Following wash with PBS, bound sE2 levels were analyzed by immunoblot assay. We showed that protein levels of sE2 bound on cells surface were decreased by O859585 in a dose-dependent manner (Fig. 3B). At 40 µM concentration, O859585 exhibited the similar level of neutralizing activity as CD81 Ab (Fig. 3B, lane 3 versus lane 4). Using GFP-tagged CD81, we further demonstrated that O859585 markedly disrupted protein association between sE2 and CD81 (Fig. 3C). These data suggest that O859585 may block HCV entry by interrupting protein interaction between viral E2 and host CD81.

### O859585 exhibits no effect on HCV internal ribosome entry site (IRES)-mediated translation, replication, and virion production

**Figure 4:**
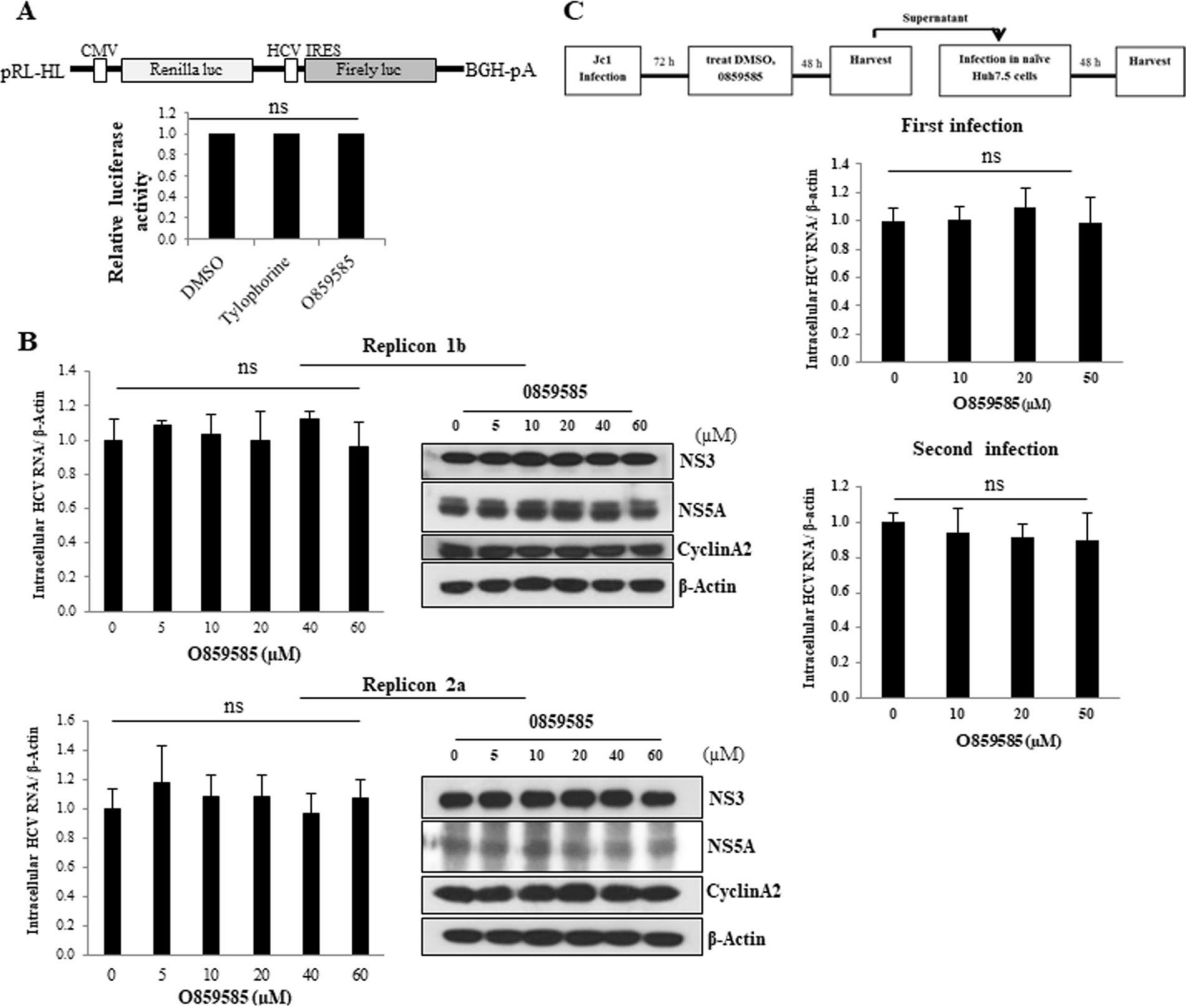
O859585 displays no effect on IRES-mediated HCV translation, replication, and virion production. (**A**) A schematic diagram of the pRL-HL plasmid is shown on top. Huh7.5 cells were treated with 0.1% DMSO, 0.075 µM tylophorine or 20 µM O859585. At 1 h after treatment, Huh7.5 cells were transfected with pRL-HL dual luciferase and a pCH110 β-galactosidase plasmid. At 48 h after transfection, relative luciferase activity was determined. (**B**) Huh7 cells harboring HCV subgenomic replicon derived from either 1b (upper) or 2a (lower) were treated with either 0.1% DMSO or increasing doses of O859585. At 48 h after treatment, viral RNA and proteins expression levels were analyzed by qRT-PCR and immunoblot assays, respectively. (**C**) Schematic diagram of the experimental set-up is shown on top. (Upper) Huh7.5 cells were infected with Jc1. At 72 h postinfection, cells were treated with various concentrations with O859585. At 48 h after treatment, intracellular HCV RNA level was analyzed by qRT-PCR. (Lower) Naïve Huh7.5 cells were infected with Jc1 containing culture supernatant harvested from the first infection. At 48 h postinfection, intracellular HCV RNA level was quantified by qRT-PCR. Data are means of 3 independent experiments.

We further explored the possible inhibitory effect of O859585 on other steps of the HCV life cycle. We first investigated whether O859585 inhibited HCV IRES-mediated translation. For this purpose, Huh7.5 cells treated with the indicated chemicals were transfected with pRL-HL dual luciferase and a pCH110 β-galactosidase plasmid, and then luciferase activity was determined as we reported previously^14^. Figure 4A demonstrated that O859585 displayed no effect on HCV IRES-mediated translation. We then analyzed the inhibitory effect of O859585 on HCV replication in Huh7 cells harboring HCV subgenomic replicon derived from either genotype 1b or 2a^18^. As shown in Fig. 4B, neither intracellular HCV RNA nor HCV protein levels were altered by O859585 in both HCV replicons. It has been previously reported that tylophorine interferes the cell cycle through modulating Cyclin A2^8^. However, we showed that O859585 did not modulate Cyclin A2 protein level (Fig. 4B). To further investigate whether O859585 inhibited virion production of HCV, Huh7.5 cells infected with Jc1 for 3 days were treated with O859585 for 48 h. As shown in Fig. 4C, intracellular HCV RNA levels were not affected by O859585 (upper panel). To verify these results, naïve Huh7.5 cells were infected with Jc1 containing culture supernatant harvested from the first infection. Consistently, O859585 displayed no discernible effect on viral infectivity (Fig. 4C, lower panel). These data suggest that O859585 mainly targets the entry step but not replication, translation, and virion production stages of the HCV life cycle.

### O859585 displays no effects on interferon signaling pathway

**Figure 5:**
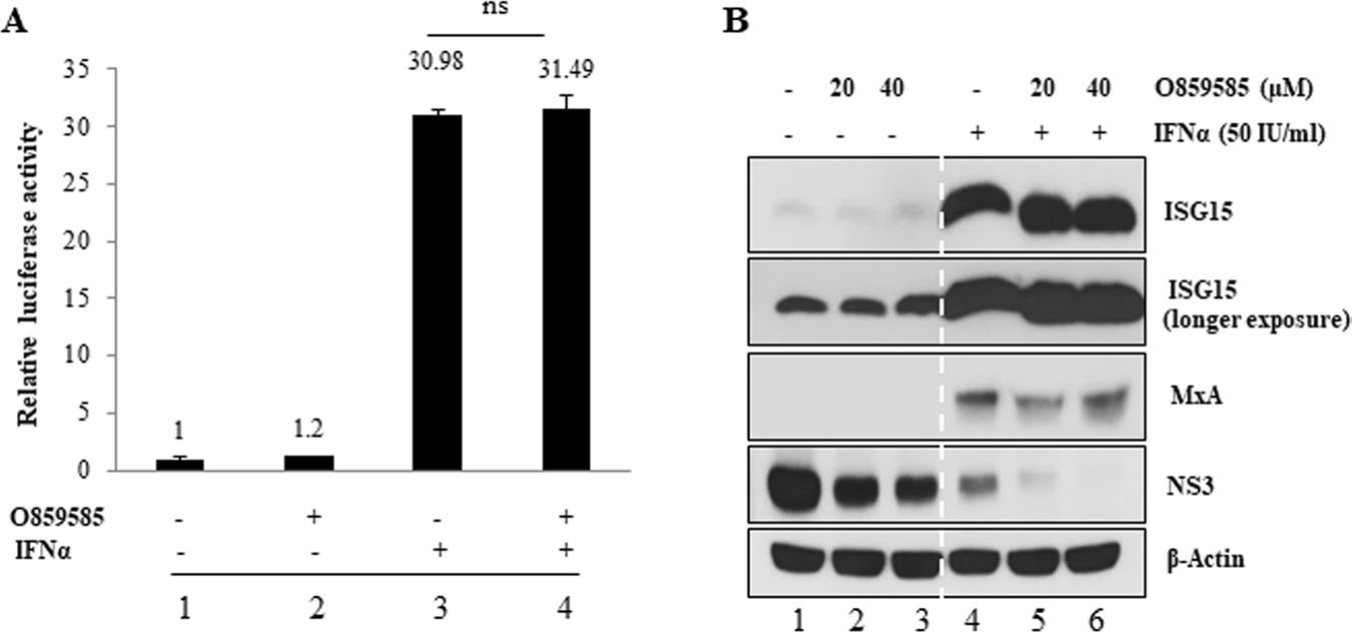
O859585 exerts no IFNα stimulation. (**A**) Huh7.5 cells were transfected with a reporter plasmid carrying IFN sensitive responsive elements (ISRE) upstream of the luciferase gene and then either left untreated or treated with 20 μM O859585, 50 IU/mL IFNα, individually or co-treated with O859585 and IFNα for 24 h and then luciferase activity was determined. Experiments were performed three times. ns, non-significant. (**B**) Huh7.5 cells infected with Jc1 were treated with the indicated combinations of O859585 and IFNα. At 48 h after treatment, total cell lysates were immunoblotted with the indicated antibodies.

IFNα represses HCV infection through activation of IFN-sensitive response elements (ISRE) which mediate transcription of downstream IFN-stimulated genes (ISGs) such as ISG15, OAS1, and MxA^19,20^. Moreover, various chemical compounds can induce the interferon signaling. Imidazoquinoline compounds, including resiquimod (R848) and imiquimod (R837), are well-known inducers of the IFN response that bind TLR7, TLR8 or both^21^. To investigate whether O859585 exerted IFN response, Huh7.5 cells were transfected with a reporter plasmid carrying ISRE and then treated with O859585. As shown in Fig. 5A, IFN significantly increased ISRE-mediated transcription activity, whereas O859585 exerted no effects on ISRE-mediated transcription activity. Moreover, IFN-induced transcription activity was not further increased by O859585 (lane 3 versus lane 4). Consistently, O859585 displayed no effect on protein expression levels of ISG15 and MxA (Fig. 5B, lane 4 versus lanes 5 and 6). These data suggest that O859585 inhibits HCV entry in an IFN-independent manner.

### O859585 in combination with IFNα and sofosbuvir exhibits strong anti-HCV activity

**Figure 6:**
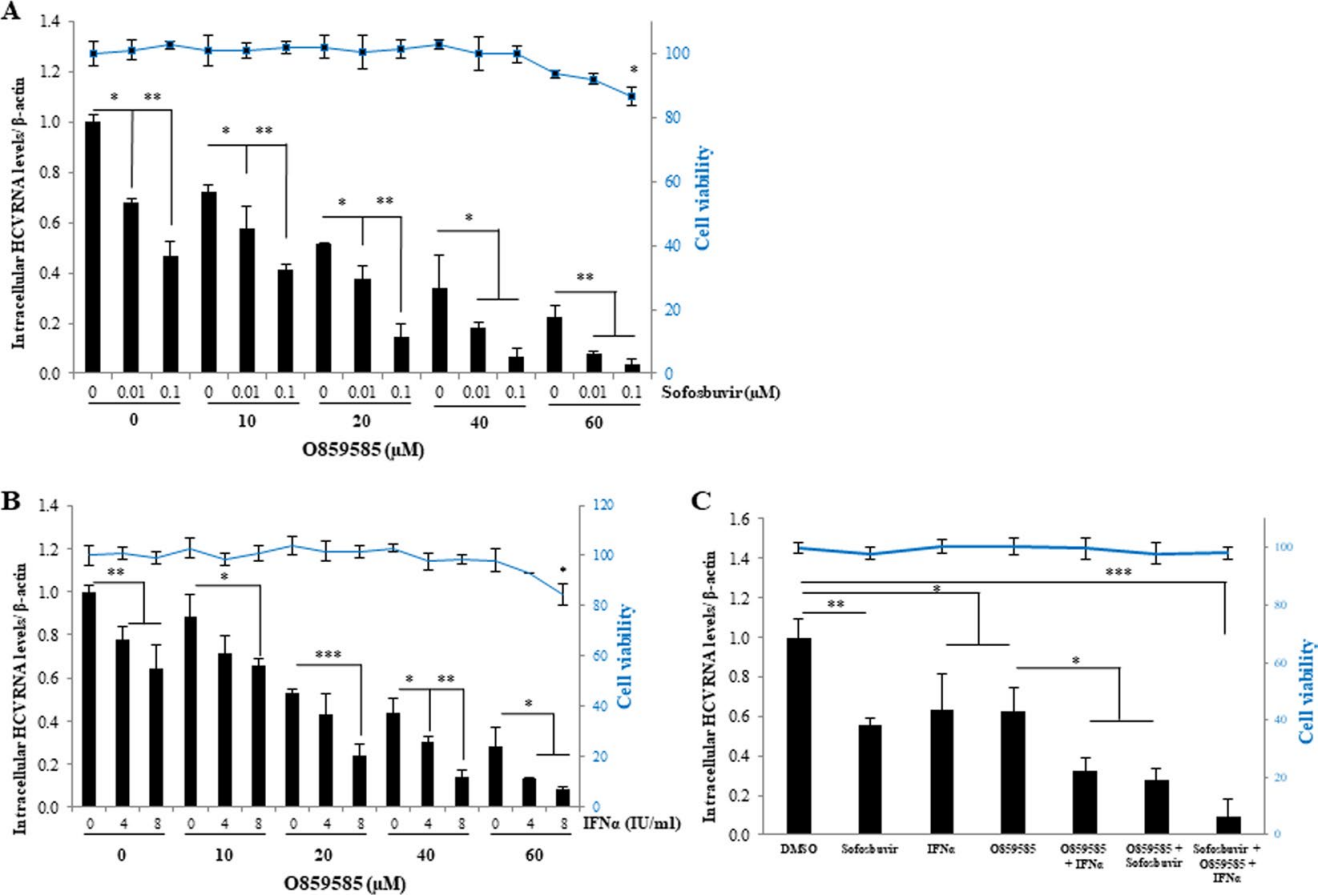
O859585 in combination with IFNα and sofosbuvir exhibits an additive anti-HCV activity. (**A**) Huh7.5 cells were infected with Jc1 in the presence of various concentrations of O859585 for 4 h. Culture medium was replaced with fresh medium containing the indicated concentrations of sofosbuvir. At 48 h postinfection, both intracellular HCV RNA level (left) and cell viability (right) were analyzed by qRT-PCR and WST assay, respectively. (**B**) Huh7.5 cells were infected with Jc1 in the presence of various concentrations of O859585 for 4 h. Culture medium was replaced with fresh medium containing the indicated concentrations of IFNα. At 48 h postinfection, both intracellular HCV RNA level (left) and cell viability (right) were analyzed by qRT-PCR and WST assay, respectively. (**C**) Primary human hepatocytes infected with Jc1 were treated with the indicated combinations of 20 µM O859585, IFNα, and sofosbuvir. At 48 h postinfection, both intracellular HCV RNA level (left) and cell viability (right) were analyzed by qRT-PCR and WST assay, respectively. Data represent means ± SD of three independent experiments. *P*-values are indicated by asterisks (*p < 0.05; **p < 0.01; ***p < 0.001).

To explore the feasibility of using the O859585 in combination with currently available HCV drugs, Huh7.5 cells infected with Jc1 were treated with various concentrations of O859585 in combination with sofosbuvir. As shown in Fig. 6A, intracellular HCV RNA levels were significantly decreased by sofosbuvir in a dose-dependent manner. Of note, sofosbuvir-mediated anti-HCV activity was significantly increased by O859585 without causing cytotoxicity. We also demonstrated that IFNα-induced suppression of HCV RNA levels were significantly increased by O859585 (Fig. 6B), indicating that supplementation of O859585 led to a stronger reduction of HCV infectivity. To verify these results, we quantified the combination effects of O859585 with either sofosbuvir or IFNα using isobolograms as reported previously^22^. The combination index (CI) value of O859585 (20 µM) and IFNα (4 IU/ml) was 0.95 and thus combination effect of O859585 and IFNα shows additivity, whereas the CI value of O859585 (10 µM) and sofosbuvir (0.1 µM) was 0.68, indicating the synergistic combination effect^22^. To further investigate combination effects of O859585 with other HCV DAAs, we selected daclatasvir (NS5A inhibitor) and asunaprevir (NS3/4A inhibitor). As shown in Supplementary Fig. S1, CI value of O859585 (10 µM) and daclatasvir (0.1 nM) was 0.78, indicating that combination effect shows synergy. Moreover, CI value of O859585 (10 µM) and asunaprevir (10 nM) was 0.52, indicating that combination effect was also synergistic (Supplementary Fig. S2). We further investigated the effect of O859585 on triple combination therapeutics. For this purpose, primary human hepatocytes infected with Jc1 were treated with the indicated combination of O859585, IFNα, and sofosbuvir. As shown in Fig. 6C, either sofosbuvir or IFNα alone exerted significant anti-HCV activity. We further showed that O859585 in combination with either IFNα or sofosbuvir exhibited an additive anti-HCV activity. Most importantly, triple combination of O859585, IFNα, and sofosbuvir exhibited the strongest antiviral activities by decreasing 90% HCV RNA level without inducing cellular toxicity (Fig. 6C). These data suggest that O859585 may be a potent candidate for combination therapy for HCV infection.

## Discussion

Chronic infection of HCV can lead to cirrhosis and HCC. HCV patients are now treatable with the advent of numerous DAAs. However, these antivirals still show genotypic difference in cure rate and have some resistance-associated variants. Moreover, these drugs are so expensive that it may limit access to the majority of HCV patients. Taking these considerations into account, it is imperative to discover novel cost-effective drug candidates for HCV patients. Several compounds, including (4R,6S)-2-Dihydromenisdaurilide^1^, Micrococcin P1^13^, (−)-epigallocatechin-3-gallate^23^, and trihydroxybenzoic gallic acid^24^ have been demonstrated to inhibit HCV entry. It has been previously reported that tylophorine isolated from *Tylophora indica* plants has an unique architecture known as the phenanthroindolizidine alkaloid and exhibits a potent growth inhibitory effect against all 60 human–derived cancer cell lines, especially HCC and HCV infection^9,11,25^. Tylophorine synthesis was first commenced with the decarboxylation of T298875 to the substituted phenanthrene^26^. Jin *et al*. also synthesized O859585, followed by ketonization process to secure tylophorine^27^. Both T298875 and O859585 are vital compounds to synthesize tylophorine. We report here for the first time that O859585 efficiently inhibits HCV entry without inducing cellular cytotoxicity.

Both O859585 and T298875 are precursors of tylophorine. In the present study, we showed that both chemicals exerted strong anti-HCV activities without causing cellular toxicity. Indeed, cell proliferation was not altered at high dosages of both chemicals. Because O859585 exerted stronger antiviral activity than T298875 at lower working concentration, we selected O859585 for further characterization. We then verified the anti-HCV activity of O859585 in HCV-infected primary human hepatocytes. We subsequently claimed that O859585 worked as a potent antagonist of HCV genotype 1a (H77D) and 2a (JFH1), which are the most popular genotypes worldwide^2^. Of note, anti-HCV activity of O859585 was higher in genotype 1a than genotype 2a, indicating that O859585 exhibited genotypic difference in antiviral activity. We determined that the EC_50_ of O859585 ranged from 29.11 µM to 35.35 µM among viral genotypes.

To investigate action mechanism of O859585 against HCV, Huh7.5 cells were pre-treated, co-treated, or post-treated with chemicals in terms of HCV infection. Here, we showed that O859585 inhibited HCV infection if the chemicals were treated at the same time of HCV infection. However, neither pre-treatment nor post-treatment of chemicals failed to inhibit HCV infection. We further demonstrated that O859585 specifically impaired HCV entry without affecting HCV replication, HCV IRES-mediated translation, and virion production. We further verified that O859585 blocked HCVpp entry but not VSV entry, indicating that anti-viral activity of O859585 was specific to HCV. The viral entry steps comprise of binding–attachment, clathrin-mediated endocytosis, and membrane fusion^28^. We clearly showed that O859585 impaired binding and attachment of HCV derived from both HCVcc and HCV-LP. At an attachment step, HCV E2 plays a key role in interaction with cellular tetraspanin molecule CD81, scavenger receptor class B type 1 (SRB1), and tight junction proteins to facilitate viral infection^29^. Because O859585 exerts a strong antiviral activity at binding/attachment step of the entry, it is plausible that O859585 likely targets viral glycoprotein E2, leading to conformational change. This in turn obstructs its receptor interaction rather than direct blocking of the cellular receptors. This hypothesis was supported by the fact that O859585 inactivated free viral particles and negatively affected binding affinity of E2 onto CD81. To further determine whether O859585 was virucidal, we separated virus from drug by using size exclusion spin column and added directly to the naïve Huh7.5 cells. As shown in Supplementary Fig. S3, O859585 appeared to have virucidal activity.

Although O859585 solely exerted profound anti-HCV activity, we explored the possible role of O859585 in combination therapy for HCV patients. For this purpose, Huh7.5 cells infected with Jc1 were co-treated with either O859585 and sofosbuvir, or O859585 and IFNα. We showed that O859585 exerted an additive effect on anti-HCV activity in combination with either sofosbuvir or IFNα. We demonstrated that the additive antiviral effect of O859585 was markedly higher in combination with sofosbuvir than in combination with IFNα. Using primary human hepatocytes as an experimental model, we further verified an additive effect of O859585 on anti-HCV activity in combination with either sofosbuvir or IFNα. Most importantly, triple combination of O859585/sofosbuvir/IFNα exerted the highest anti-HCV activity in a synergistic fashion. Since the main target of O859585 is the entry of HCV infection, O859585 may be considered as a potential therapeutic candidate for liver transplantation, a primary option for curing end-stage liver disease associated with HCV. It has been previously reported that HCV reinfection in allograft is inevitable after transplantation, and treatment with PegIFNα plus ribavirin or neutralizing antibodies is almost ineffective^29–31^. We therefore postulate that O859585 may be a potential candidate to overcome this issue. Taken together, our study demonstrated that O859585 could be employed as a combinatorial therapeutic agent for the treatment of HCV infection.

## Methods

### Cell culture

Huh7.5 cells were cultured in Dulbecco’s modified Eagle’s medium (DMEM) supplemented with 10% fetal bovine serum (FBS), 1% nonessential amino acids, and 1% penicillin-streptomycin in 5% CO_2_ at 37 °C. HEK293T cells were grown in DMEM containing 5% FBS. Huh7 cells harboring HCV subgenomic replicon derived from genotype 1b or 2a, and IFN-cured cells were grown as reported previously^18^. Primary human hepatocytes (Invitrogen) were cultured as we reported previously^14^.

### Antibodies and reagents

Antibodies were purchased from the following sources: anti-CD81 antibody was from BD Pharmingen, mouse anti-actin antibody was from Sigma-Aldrich, and ISG15 and MxA antibodies were from Santa Cruz. HCV core, NS3, NS5A antibodies have been described elsewhere^14^. HCV E2 antibody was a gift from Dr. Jean Dubuisson (Institut Pasteur de Lille). Tylophorine was purchased from Alexis Biochemicals. O859585, T298875, and Sofosbuvir were purchased from Toronto Research Chemicals and MedChemexpress.

### Preparation of infectious HCV

HCVcc was generated as described previously^18^. Briefly, the monolayer of Huh7.5 cells was washed twice in PBS, trypsinized, and resuspended at a concentration of 5 × 10^6^ cells/ml in Opti-MEM (Invitrogen, Carlsbad, CA). After centrifugation at 850 rpm for 3 min, the cells were resuspended in 360 µl of cytomix solution containing 2 mM ATP and 5 mM glutathione. The cells were then mixed with 10 µg of Jc1 RNA and were electroporated using the Gene Pulser Xcell (Bio-Rad Laboratories, Hercules, CA) in a 4-mm gap cuvette. Cell culture supernatants were collected at 4 days after electroporation using a 0.45 µm syringe.

### Western blot analysis

Cells were washed twice with cold PBS and lysed in radioimmunoprecipitation assay (RIPA) buffer containing 20 mM Tris HCl (pH 7.2), 150 mM NaCl, 1% NP-40, 10 mM NaF, 30 mM sodium pyrophosphate, 1 mM EDTA, 1 mM Na_3_VO_4_, 1 mM phenylmethylsulfonyl fluoride (PMSF), and protease inhibitor cocktail for 15 min on ice. The samples were centrifuged at 13,000 rpm for 10 min at 4 °C. The supernatant was collected and protein concentration was determined by the Bradford assay kit (Bio-Rad). Equal amounts of protein were subjected to SDS-PAGE and electrotransferred to a nitrocellulose membrane. The membrane was blocked in Tris-buffered saline (TBS)-Tween (20 mM Tris-HCl (pH 7.6), 150 mM NaCl, and 0.25% Tween 20) containing 5% nonfat dry milk for 1 h. The membrane was further incubated with the indicated primary antibodies and followed by either rabbit or mouse secondary antibodies for 1 h at room temperature. Proteins were detected using an ECL kit (Abfrontier).

### Quantification of RNA

Total RNAs were isolated from cells using RiboEx LS reagent (Geneall Biotechnology) according to the manufacturer’s instructions. cDNAs were synthesized by using a cDNA synthesis kit (Toyobo). Quantitative real-time PCR (qRT-PCR) experiments were performed using a CFX Connect real-time system (Bio-Rad Laboratories, Hercules, CA) with following primers: HCV-specific primers (sense, 5′-TTA GTA TGA GAG TCG TAC AGC CTC CAG-3′; antisense, 5′-GGC ATA GAG TGG GTT TAT CCA AGA AAG G-3′), β-actin-specific primers (sense,5′-TGA CAG CAG TCG GTT GGA GCG-3′; antisense, 5′-GAC TTC CTG TAA CAA CGC ATC TCA TA-3′) as we reported previously^14,18^.

### WST assay

Approximately 4 × 10^4^ cells/well seeded on 24-well plates were treated with the indicated chemicals. At the indicated time points, water-soluble tetrazolium salt (WST) (Dail Lab) was added and incubated at 37 °C for 1 h. Cell viability was determined as we reported previously^14^.

### Immunofluorescence assay

Huh7.5 cells were seeded on glass coverslips and treated with 0.1% DMSO, 0.075 µM tylophorine, 20 µM T298875, and 20 µM O859585, respectively for 1 h. Cells were infected with Jc1 for 4 h in the presence of each chemical. At 48 h postinfection, cells were washed twice with PBS and fixed with 4% paraformaldehyde in PBS for 10 min and then permeabilized with 0.1% Triton X-100 for 10 min at room temperature. After three washes with PBS, fixed cells were blocked with 0.25% BSA in PBS. Cells were then incubated with NS5A antibody at 4 °C overnight. After three washes with PBS, cells were further incubated with tetramethylrhodamine isothiocyanate (TRITC)-conjugated goat anti-mouse IgG at room temperature for 1 h. Cells were counterstained with 4′,6-diamidino-2-phenylindole (DAPI) to label nuclei. After three washes with PBS, cells were analyzed using the Zeiss LSM 700 laser confocal microscopy system (Carl Zeiss, Inc., Thornwood, NY).

### Time-of-addition assay

Time-of-addition assay was performed as described previously^32^. Briefly, 0.7 × 10^3^ Huh7.5 cells/well were seeded on 12-well plates and incubated for 24 h. Cells were treated with 0.1% DMSO, 20 µM O859585, 5 µM erlotinib, respectively as follows. Pretreatment: Huh7.5 cells were treated with chemicals at 4 h before HCV infection. Co-treatment: HCV mixed with chemicals was inoculated to Huh7.5 cells. Post-entry treatment: HCV-infected cells were treated with chemicals at 4 h postinfection. At 2 days postinfection, total RNA levels were analyzed by qRT-PCR.

### HCV pseudoparticle entry assay

HCV pseudoparticles (HCVpp) with E1 and E2 glycoproteins derived from genotype 1a (H77D) or genotype 2a (JFH1) and vesicular stomatitis virus pseudoparticles (VSVpp) were generated as we reported previously^14^. Briefly, HEK293T cells were transfected with either HCV E1E2 plasmid or VSV G envelope expressing plasmid, Gag-Pol (polymerase) packing plasmid, and transfer vector encoding the firefly luciferase reporter protein by using polyethyleneimine (Sigma- Aldrich). At 48 h after transfection, supernatants containing HCVpp or VSVpp were collected. Subsequently, Huh7.5 cells were treated with 0.1% DMSO, 20 µM O859585, 5 µM erlotinib, respectively and then infected with either HCVpp or VSVpp for 6 h. Cells were then replaced with fresh culture media. At 48 h postinfection, cells were harvested and luciferase activity was determined.

### Soluble E2 (sE2) binding and neutralization assays

For sE2 binding assay, HEK293T cells were transfected with plasmid encoding sE2 derived from H77D using polyethyleneimine in serum-free media and incubated for a further 48 h with media containing 10% FBS^16,17,33^. The supernatant containing sE2 was harvested and condensed by Amicon Ultra-4 Centrifugal Filter Unit (Merck, Germany) and sE2 was further purified as we reported previously^34^. Huh7.5 cells were incubated with sE2 in the presence of various concentrations of 0.1% DMSO, O859585, and 1 µg/ml anti-CD81 antibody, respectively for 4 h. Unbound proteins were removed by washing with cold PBS and bound sE2s were detected by Western blot assay. For free viral particle neutralization assay, virus and chemicals were mingled for 3 h at 37 °C. The mixtures were diluted to 20 times in order to deactivate chemicals. Monolayer of Huh7.5 cells was infected with virus containing the diluted chemicals for 4 h. Viral RNA and proteins were determined by either qRT-PCR or Western blot.

### *In vitro* drug combination assay

For *in vitro* drug combination assay, either primary human hepatocytes or Huh7.5 cells infected with Jc1 were treated with several combinations of O859585, IFNα, and sofosbuvir for 4 h as previously described^35^. Anti-HCV infectivity of each drug alone or combinatorial drugs were analyzed by qRT-PCR. Cell viability for each drug alone or for the test combination was determined by WST assay.

### Luciferase reporter assay

Huh7.5 cells were transfected with a reporter plasmid carrying interferon sensitive response element (ISRE) upstream of the luciferase gene for 24 h and then either left untreated or treated with 20 μM O859585, 50 IU/mL IFNα, respectively or cotreated with both O859585 and IFNα. At 24 h after treatment, luciferase activity was analyzed as previously described^35^.

### Determination of combination index (CI)

The isobologram analysis evaluates the nature of interaction of two drugs, i.e., drug A and drug B, at an indicated effect level. Combination index (CI) is calculated by the equation as reported previously^22^.

$$CI=\frac{{C}_{A,x}}{I{C}_{x,A}}+\frac{{C}_{B,x}}{I{C}_{x,B}}$$

The concentrations required to produce the given effect (e.g., IC50) are determined for drug A (IC_x,A_) and drug B (IC_x,B_) and indicated on the x and y axes of a two-coordinate plot. The concentrations of A and B contained in combination that provide the same effect, denoted as (C_A,x_, C_B,x_). A CI of less than, equal to, and more than 1 indicates synergy, additivity, and antagonism, respectively.

### Statistical analysis

Data analyzed were presented as means ± standard deviations (SDs) from three independent experiments. Student *t* test was used for statistical analysis. The asterisks on the figures indicate significant differences, as noted in the figure legends.

## Supplementary information

Supplementary Data
