# Supplementary material for: 5-Oxo-1-[(2,3,6,7-tetramethoxy-9-phenanthrenyl)methyl]-L-proline Inhibits Hepatitis C Virus Entry
Source: Sci Rep. 2019 May 13;9:7288. doi: 10.1038/s41598-019-43783-6 (PMC6514212; doi:10.1038/s41598-019-43783-6)

**5-Oxo-1-[(2,3,6,7-tetramethoxy-9-phenanthrenyl)methyl]-L-proline Inhibits Hepatitis C Virus Entry**

**Lap P. Nguyen†^1,2^, Chorong Park†^2^, Trang T. D. Luong^2^, Eun-Mee Park^3^, Dong-Hwa Choi^4^, Kang Min Han^5^, Han N. Mai ^1,2^, Huu C. Nguyen ^1^, Yun-Sook Lim^1,2,*^, Soon B. Hwang^1,2,*^**

^1^Laboratory of RNA Viral Diseases, Korea Zoonosis Research Institute, Chonbuk National University, Iksan, South Korea, ^2^National Research Laboratory of Hepatitis C Virus, Hallym University, Anyang, South Korea, ^3^Korea National Institute of Health, Cheongju, South Korea, ^4^Graduate School of East-West Medical Science, Kyung Hee University, Yongin, South Korea, ^5^Department of Pathology, Dongguk University Ilsan Hospital, Goyang, South Korea

**Supplementary Figure Legends**

**Figure S1** **O859585 in combination with asunaprevir exhibits a synergistic anti-HCV activity.** Huh7.5 cells were infected with Jc1 for 4 h in the presence of various concentrations of O859585. Culture medium was replaced with fresh medium containing the indicated concentrations of asunaprevir. At 48 h postinfection, HCV RNA levels were analyzed by qRT-PCR. Data represent means ± SD of two independent experiments. *P*-value is indicated by asterisk (*, p < 0.05).

**Figure S2 O859585 in combination with daclatasvir exhibits a synergistic anti-HCV activity.** Huh7.5 cells were infected with Jc1 for 4 h in the presence of various concentrations of O859585. Culture medium was replaced with fresh medium containing the indicated concentrations of daclatasvir. At 48 h postinfection, HCV RNA levels were analyzed by qRT-PCR. Data represent means ± SD of two independent experiments. *P*-value is indicated by asterisk (*, p < 0.05).

**Figure S3 O859585 has limited virucidal activity.** Jc1 virions were mingled with either DMSO or various concentrations of O859585 for 2 h at 37°C. The mixture was centrifuged for 15 min at 2000 rpm using the Amicon® Ultra-15 Centrifugal Filter Units to eliminate unbound O859585. The virus-O859585 mixture was resuspended in 5 ml of fresh DMEM. Naïve Huh7.5 cells were infected with diluted mixture containing Jc1. At 48 h postinfection, protein levels were analyzed by immunoblot analysis using the indicated antibodies.

Supplementary Figure S1


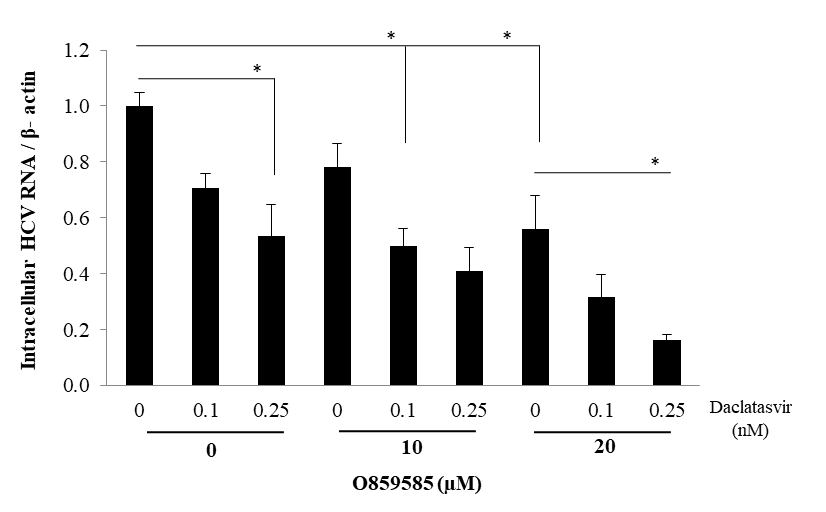


Supplementary Figure S2


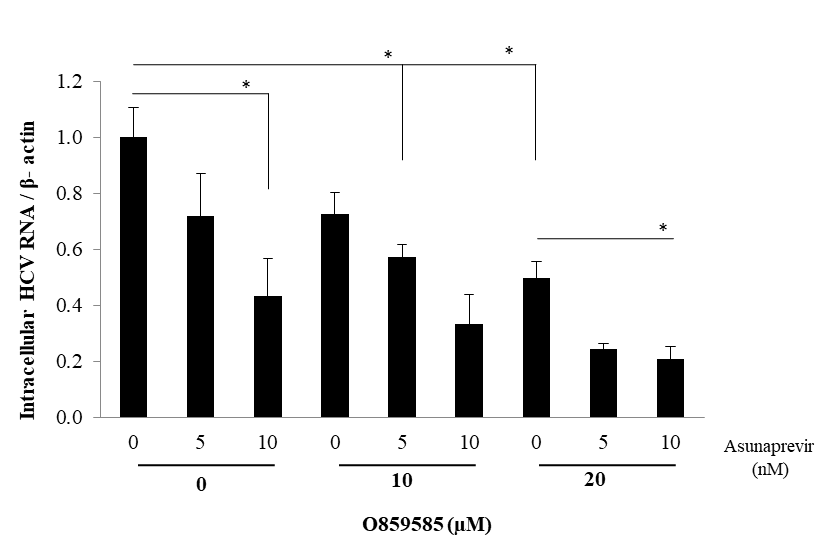


Supplementary Figure S3


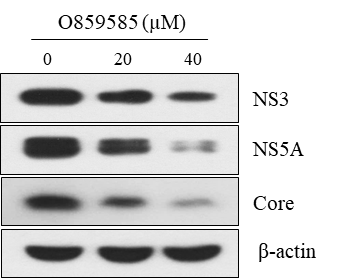

Supplement: Supplementary file 1 — Supplementary Data [file 41598_2019_43783_MOESM1_ESM.doc]
